# Pavement crack analysis by referring to historical crack data based on multi-scale localization

**DOI:** 10.1371/journal.pone.0235171

**Published:** 2020-08-14

**Authors:** Xianglong Wang, Hu Zhaozheng, Na Li, Lingqiao Qin

**Affiliations:** 1 ITS Research Center, Wuhan University of Technology, Wuhan, China; 2 School of Automation, Wuhan University of Technology, Wuhan, China; 3 TOPS Laboratory, Department of Civil and Environmental Engineering, University of Wisconsin-Madison, Madison, WI, United States of America; Beijing University of Technology, CHINA

## Abstract

Pavement crack analysis, which deals with crack detection and crack growth detection, is a crucial task for modern Pavement Management Systems (PMS). This paper proposed a novel approach that uses historical crack data as reference for automatic pavement crack analysis. At first, a multi-scale localization method, which including GPS based coarse localization, image-level localization, and metric localization has been presented to establish image correspondences between historical and query crack images. Then historical crack pixels can be mapped onto the query crack image, and these mapped crack pixels are seen as high-quality seed points for crack analysis. Finally, crack analysis is accomplished by applying Region Growing Method (RGM) to further detect newly grown cracks. The proposed method has been tested with the actual pavement images collected in different time. The F-measure for crack growth is 88.9%, which demonstrates the proposed method has an ability to greatly simplify and enhances crack analysis result.

## 1. Introduction

Modern Pavement Management Systems (PMS) are playing more and more important roles in pavement survey, maintenance, and rehabilitation. An increasing number of transportation agencies are building and upgrading their PMS to enhance pavement management. In PMS, pavement crack analysis, which deals with crack detection and crack growth detection, is a crucial and core task. With the development of sensor and information technology, crack data can be quickly and automatically collected by vehicle-borne sensors. For example, many transportation agencies are routinely (e.g., quarterly, semi-annually, annually, etc) using sensor vehicles for pavement image collection. Thereby, how to accurately identify pavement cracks from the collected pavement images is becoming a key technology in automatic crack analysis in PMS.

For the past decades, pavement crack detection and recognition have been extensively investigated. Generally, existing crack detection methods can be classified into two main categories: 1) 2D image data based methods; 2) 3D range data based methods. In these researches, the image-based methods are the mainstream methods, which can also be classified into two main sub-categories: supervised methods and unsupervised methods. The supervised methods are based on the supervised learning approaches that consist of the two steps of training and testing. In the training step, the image patches with cracks are labelled manually as positive samples while those patches with no cracks as negative ones. From the training data, a classifier can be trained by using different machine learning models. Finally, the trained classifier can be used for crack detection, also known as testing step. In the literature, many machine learning models are proposed to train the classifiers. For example, Chu et al applied Back Propagation (BP) neural network to train a crack detector [1]. Another important progress for supervised crack detection is the use of Convolutional Neural Network (CNN), also known as deep learning. The CNN based methods try to utilize a deep neural network with more layers to train the data and finally derive a more accurate and robust classifier [2–4]. For example, Wang et al first applied the CNN for crack detection [5]. In their work, a deep CNN of 7 layers was utilized to train a crack detector. Moreover, the authors compared two kinds of scale grid (32×32, 64×64) to achieve a good performance in crack detection. This method can achieve 95.9% precision and 93.5% recall, which demonstrates good potential of deep learning for crack analysis. Li et al utilized CNN to classify the pavement crack. In their work, the accuracy of pavement crack classification is more than 94% [6]. However, the disadvantage of the supervised methods is that a large number of labelled data is required for the training. In contrast, the unsupervised based methods require no training data. They try to segment the crack areas by using some prior knowledge of the crack areas, such as image intensity, edge, texture, etc. Many algorithms have been developed by adaptively setting the thresholds for direct crack segmentation. For example, Fujita et al utilized a locally adaptive threshold to segment crack pixels in the image. It achieved a good performance in pavement crack detection [7]. Another type of crack detection algorithms are based on seed points, which are usually selected from pavement images by comparing a pixel value within its surrounding pixels or by computing the image edges. With the seed points, the remaining crack areas can be derived by using region growing or by path optimization. For example, Kaul et al formulated the crack detection into a shortest path problem based on the initial seed point detection results [8]. As asphalt pavement images are highly textured, many texture based methods are developed for crack detection. For example, the Gabor filters have been extensively investigated for crack detection by analysing the pavement texture with a bank of filters with different scales and angles [2, 3, 9–12]. Besides Gabor filter, Li et al also proposed a steerable matched filter to extracted crack saliency map [13]. The crack saliency map can be used as a coarse initial guess to fill into an active contour method to extract all the crack areas. Besides image-based crack detection, crack detection methods based on 3D data are also developed in existing literature recently. 3D data especially laser has unique character. Many researchers have developed pavement crack detection algorithm utilizing laser scan [14–16]. For example, Tsai et al developed 3D pavement crack imaging systems, as well as the crack detection methods based on 3D data in different environment conditions [17]. The result illustrated that 3D data are more robust and reliable than image data for crack detection. In addition, since the deep learning has remarkable performance in object recognition utilizing 3D data, Zhang et al applied a deep CNN of 7 layers named CrackNet to realize crack detection through 3D data [18]. It achieved good results (Precision 90.13%, Recall 87.63%, F-measure 88.86%).

Currently, many transportation agencies are collecting pavement image data in a routinely and periodic manner. For example, most of transportation agencies collect pavement data annually, while some of them collect pavement data every 6 months. As a result, historical crack images can be obtained by several crack data collections. The pavement cracks will not grow substantially in a short period of time. Hence, the historical crack data can be utilized to enhance current crack analysis. To achieve this goal, an important task is to establish the correspondence between historical and current crack images, which is also called localization. Therefore, this paper proposed a method that using historical data as reference for current crack analysis. Especially, the proposed method aim to address the localization problem by using a multi-scale strategy. Contributions of this paper are summarized as follow: 1) The authors proposed a method that using historical crack data as the reference for pavement crack analysis, which can greatly enhance the performance of pavement crack analysis; 2) The authors proposed a multi-scale localization strategy to match historical crack image with current crack image. The multi-scale localization method consists of GPS-based coarse localization, image-level localization and finally pixel-level localization; 3) By referring to historical crack data, the authors proposed a novel approach, called RGM, it can detect the condition change of pavement cracks easily. The condition change of pavement crack is especially important for pavement treatment strategies.

The rest of this paper is organized as follow: Section 2.1 introduces the multi-scale localization and crack data mapping. Section 2.2 introduces crack detection and analysis by RGM. Section 3 presents the experimental results. Section 4 draws the conclusion.

## 2. The proposed method

**Fig 1:**
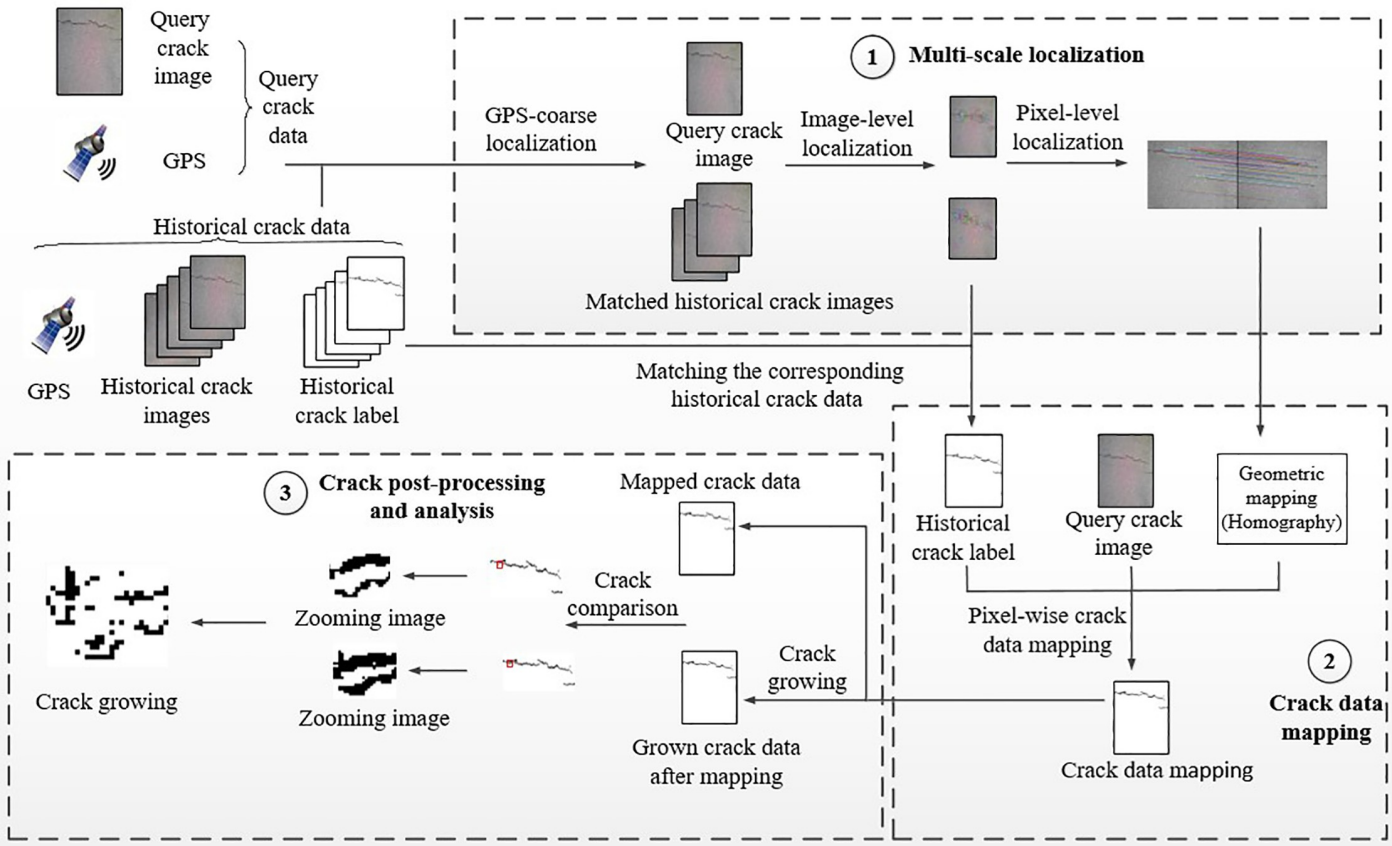
Illustration of the reference-based crack analysis method.

We got the authority of work from Wuhan university of technology. As illustrated in Fig 1, the proposed reference-based crack analysis method consists of three main modules: 1) multi-scale localization; 2) mapping historical crack image onto the query crack image; 3) crack post-processing and analysis. Each query crack data and historical crack data contains GPS information and a crack image. Each crack image is well associated with GPS information. In addition, the historical crack label, either extracted in manual or automatic way, are represented as a limited number of pixels belonging to crack (in pixel-level) in the pavement images. Hence, each historical crack data can be presented by using point sets as follows:

$$m_{i}=\left\{G_{i},I_{i},L_{i}\right\}\;(i=1,2,..,n)$$ (1)

where *n* is the number of historical crack data. *G*_*i*_ is GPS information. *I*_*i*_ is the pavement crack image and *L*_*i*_ represents all the crack pixels that are labelled in the historical image.

### 2.1. Multi-scale localization and crack data mapping

The module of multi-scale localization aims to establish the pixel-level image correspondence between the query crack data and the historical crack data. The proposed method adopts a coarse-to-fine strategy to achieve multi-scale localization. And it consists of three steps, which are GPS-based coarse localization, image-level localization and pixel-level localization. Based on the localization results, the labelled crack pixels in the historical image can be mapped onto the query crack image for crack analysis.

#### 2.1.1. GPS-based coarse localization

**Fig 2:**
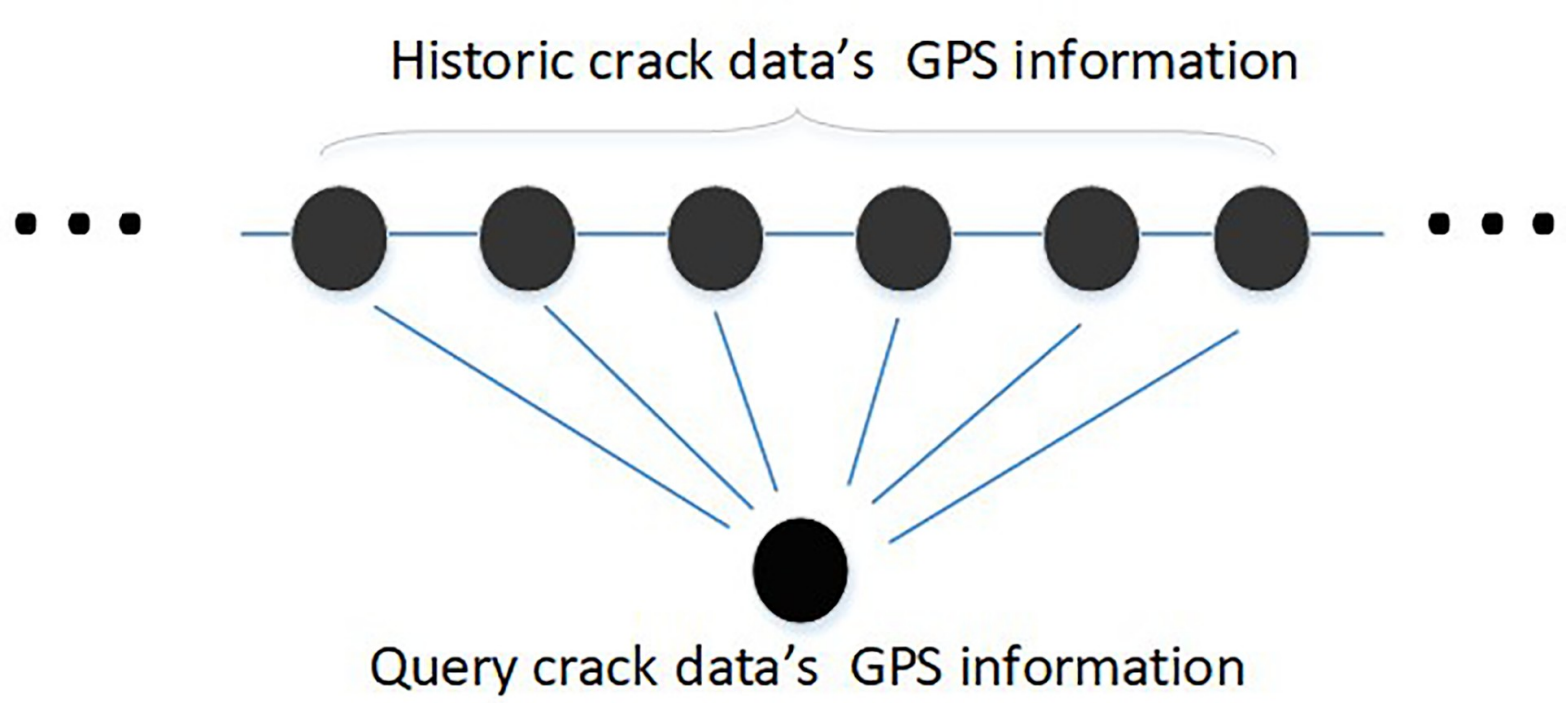
Coarse localization from GPS coordinate matching.

The first step of multi-scale localization is the coarse localization using GPS data, as illustrated in Fig 2. Let *G*_*j*_ be the GPS coordinate of *j*^*th*^ query crack data and *G*_*i*_ be the GPS coordinate of *i*^*th*^ historical crack data. The distance between *G*_*j*_ and *G*_*i*_ can be calculated as follow:

$$d_{ji}=dist\left(G_{j},G_{i}\right)$$ (2)

With the GPS data matching, a set of historical crack image candidates can be obtained, whose associated GPS coordinates and the query GPS coordinates are within a threshold distance. Thus, this step is called GPS-based coarse localization. The GPS-based coarse localization allows us to derive a limited number of candidates from a huge amount of historical crack images collected. The mathematical of this task are described as follow:

$$Pos=\{G_{i}|d_{ij}\leq k\}$$ (3)

where *k* is a threshold distance to select the candidate historical crack data. It decides that the *i*^*th*^ historical crack data is close enough to the *j*^*th*^ query crack data. *d*_*ij*_ is the distance between *i*^*th*^ historical crack data and *j*^*th*^ query crack data. In practice, according to the accuracy of GPS localization, the threshold distance is heuristically set to 10 meter. After GPS-based coarse localization, a limited number of candidate historical crack images, which satisfies Eq (2), are obtained.

#### 2.1.2. Image-level localization

The purpose of image-level localization is to find the “closest” historical crack image, which is within the candidate historical crack images from the GPS-based coarse localization results, to the query crack image. The term “closest” means that the matched historical crack image and query crack images have least distance among all the candidate historical crack images. The authors employ the local image feature point pairs, which are corresponding same local feature points for different images, to realize image-level localization. In this paper, all the images are represented with a number of local feature points. As a result, we can match two images by comparing their local features to achieve image-level localization.

In this paper, ORB [19] is utilized to extract local feature points both for query and candidate historical crack images. The ORB is an image match algorithm which combines oFAST (FAST with orientation) and rBRIEF (rotated BRIEF). Compared to the classic SIFT and SURF, ORB has much faster computation speed. More specific, it applies oFAST for feature point detection and rBRIEF for feature descriptor computation. The oFAST develops from FAST (From Accelerated Segment Test). FAST takes one parameter, the intensity threshold between the centre pixel and those in a circular window around the centre. Hence, FAST is very fast in implementation. Practically, FAST-9, which circular radius is 9, is usually used for good performance. The performance of oFAST is enhanced in two ways. First, the Harris corner measure is adopted to select the distinct FAST points. Second, the orientation information is added such that the extracted feature points are orientation invariant. The orientation is calculated from the moments of the image within a circular window:

$$m_{pq}=\sum_{x,y}x^{p}y^{q}I\left(x,y\right)\;\left(p,q\in (0,1)\right)$$ (4)

where *x*, *y* are coordinate of an image and *I*(*x*,*y*) is moment of this image. As a result, the centroid of the image is computed from the moments as follows:

$$C=\left(\frac{m_{10}}{m_{00}},\frac{m_{01}}{m_{00}}\right)$$ (5)

The orientation of the patch image is thus defined as follows:

$$\theta =atan2\left(m_{01},m_{10}\right)$$ (6)

where *atan*2 is the quadrant-aware version of *arctan*, and the BRIEF descriptor is a bit string description of an image patch constructed from a set of binary intensity tests. Consider a smooth image patch *P*, a binary test of two arbitrary positions *x* and *y* is a logic result of comparing their image intensities as follows:

$$\tau \left(P;x,y\right)=\{\begin{array}{l} 1:P\left(x\right)<P\left(y\right)\\ 0:P\left(x\right)\geq P\left(y\right) \end{array}$$ (7)

where *P*(*x*) is the intensity of the image *c* at a point *c*. The BRIEF descriptor is thus defined as a vector of *n* binary tests:

$$f_{n}\left(P;x,y\right)=\sum_{i=1}^{n}2^{i-1}\tau \left(P;x,y\right)$$ (8)

In the literature, there are many solutions on how to choose the *n* binary tests. And in this paper, the authors use a Gaussian distribution around the patch centre and choose a vector length *n* = 256. As a result, an ORB feature descriptor is represented with a 256-bit string vector. In order to make the BRIEF descriptor invariant to rotation, ORB steers BRIEF according to the orientation of the key points. For any feature set of *n* binary test, ORB defines the following 2×*n* matrix:

$$S=\left(\begin{array}{c} x_{1},\ldots ,x_{n}\\ y_{1},\ldots ,y_{n} \end{array}\right)$$ (9)

From the patch orientation *θ*, the corresponding rotation matrix *R*_*θ*_ can be computed as follow:

$$R_{\theta }=\left(\begin{array}{cc} \cos\theta & -\sin\theta \\ \sin\theta & \cos\theta \end{array}\right)$$ (10)

Then a steered version *S*_*θ*_ can constructed by rotation matrix *R*_*θ*_ and *S* as follows:

$$S_{\theta }=R_{\theta }S$$ (11)

From the steered BRIEF, the authors can compute the rotation-invariant descriptor, also known as ORB descriptor, as follows:

$$g_{n}\left(Ρ,\theta \right)=f_{n}\left(Ρ\right)|\left(x_{i},y_{i}\right)\in S_{\theta }$$ (12)

The matching of local features across different images is based on the Hamming distances. More details are referred to [19]. The query image is thus matched with all the candidate historical images by ORB-feature based matching. And the “closest” historical crack image that has the most number of matched feature points is derived as the image-level localization result.

#### 2.1.3. Pixel-level localization for historical crack mapping

Once the “closest” historical crack image is obtained, the underlying geometric relationship between the query and the historical images can be calculated by pixel-level localization. At first, the authors assume that the pavement is a plane. Therefore, under a pin-hole camera model, the underlying geometry can be described with a homography matrix [20] such that:

$$\left[\begin{array}{c} x_{i}\\ y_{i}\\ 1 \end{array}\right]≅H\left[\begin{array}{c} \mu_{i}\\ \nu_{i}\\ 1 \end{array}\right]$$ (13)

where *μ*_*i*_, *ν*_*i*_ are the coordinate of local feature on the historical image and *x*_*i*_, *y*_*i*_ are the coordinate of local feature on the query image.

$$H=\left[\begin{array}{ccc} h_{1} & h_{2} & h_{3}\\ h_{4} & h_{5} & h_{6}\\ h_{7} & h_{8} & h_{9} \end{array}\right]$$ (14)

Eq (13) can be re-written as follows:

$$\begin{array}{c} x_{i}=\frac{h_{1}\mu_{i}+h_{2}\nu_{i}+h_{3}}{h_{7}\mu_{i}+h_{8}\nu_{i}+h_{9}}\\ y_{i}=\frac{h_{4}\mu_{i}+h_{5}\nu_{i}+h_{6}}{h_{7}\mu_{i}+h_{8}\nu_{i}+h_{9}} \end{array}$$ (15)

From the above equation, the authors can generate two linear constraints on the homography matrix:

$$\left[\begin{array}{ccccccccc} \mu_{i} & \nu_{i} & 1 & 0 & 0 & 0 & -x_{i}\mu_{i} & -x_{i}\nu_{i} & -x_{i}\\ 0 & 0 & 0 & \mu_{i} & \nu_{i} & 1 & -y_{i}\mu_{i} & -y_{i}\nu_{i} & -y_{i} \end{array}\right]{\left[\begin{array}{llll} h_{i} & h_{2} & \cdots & h_{9} \end{array}\right]}^{T}=0$$ (16)

As the homography matrix can be determined up to a scale, the homography matrix can be computed from at least 4 point correspondences. In practice, the Direct Linear Transform (DLT) can be applied to compute the homography matrix and optimize the results with the Levenberg-Marquardt (LM) method. The computation details can be referred in [20].

With the computed homography matrix, the historical crack label can be mapped onto the query image as follow:

$${\left[x_{i}\;y_{i}\;1\right]}^{T}≅H{\left[\mu_{i}\;\nu_{i}\;1\right]}^{T}\;\left(i=1,2,\ldots ,n\right)$$ (17)

where *n* is the number of labelled crack pixels on historical image. [*μ*_*i*_ *ν*_*i*_]^*T*^ is the coordinates of pixel which is the mapped crack label on the historical image. [*x*_*i*_ *y*_*i*_]^*T*^ is the coordinate of mapped crack label on the query image.

As a result, the mapped crack label on the query images can be represented with a set of 2D image coordinates as follow:

$$Q=\left\{{[x_{i}\;y_{i}]}^{T}\right\}\;\left(i=1,2,\cdots n\right)$$ (18)

With Eq (18), we can determine the mapped crack pixels on the query images from pixel-level localization results.

### 2.2. Crack detection and analysis by RGM

Once the authors obtain the crack pixels on the query image by mapping the historical crack pixels, which were well labelled in the historical database, all the crack pixels can be detected afterwards. These mapped crack pixels on the query images are important clues for crack detection. The grown crack pixels of query images can be detected by RGM through mapped crack pixels.

**Fig 3:**
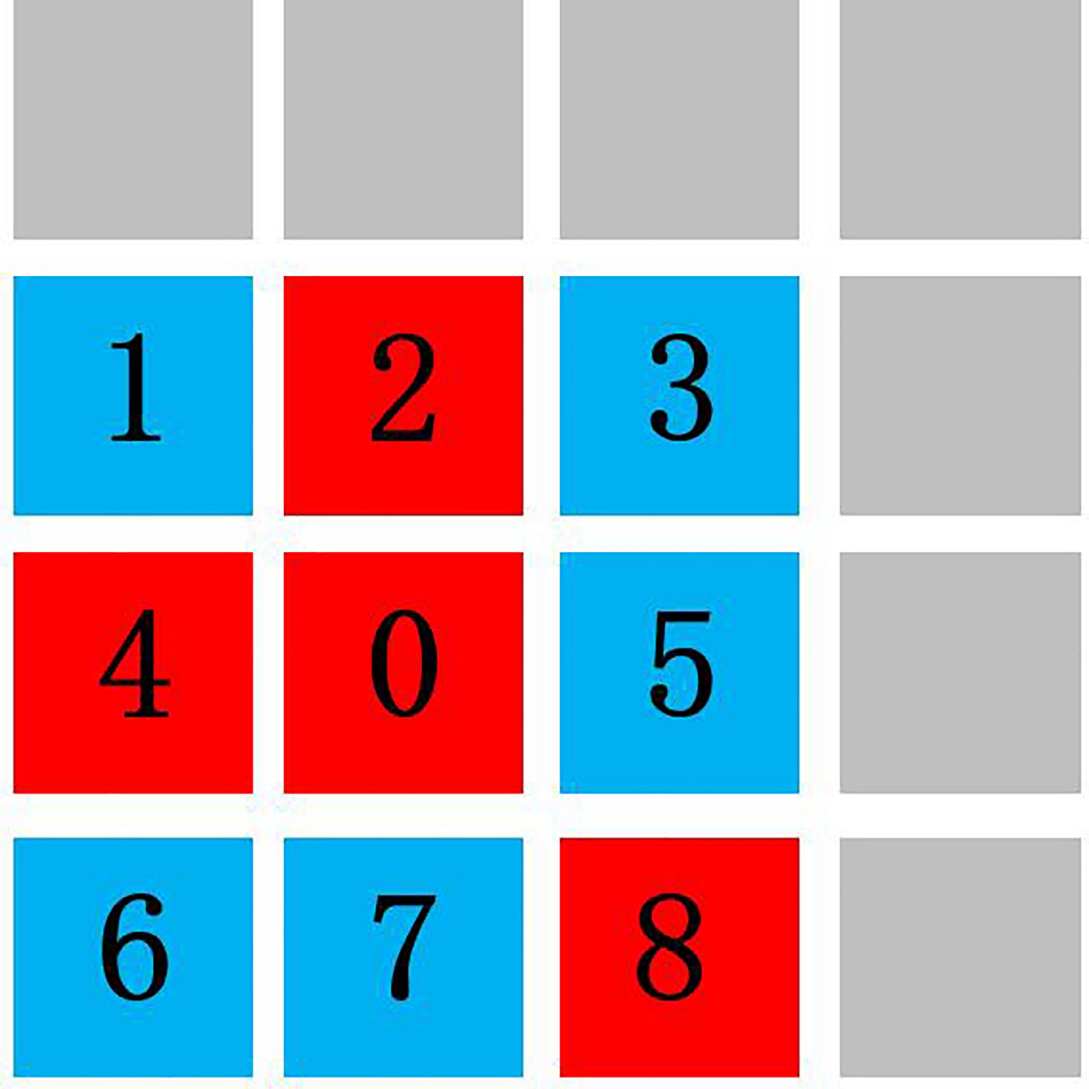
Seed point from mapping for crack region growing.

As crack situations can be deteriorate during the time interval between the current and historical crack data collection operations, there are some pixels belonging to newly grown crack that need to detect as well. This paper proposes using the RGM for crack post-processing and analysis. According to [21, 22], the newly grown crack pixel is close to existing crack pixel. The existing crack pixel, which is the mapped query crack label from the above steps, can be used as “ideal” initial seed points. The aim of RGM is to find the newly grown crack pixels according to seed points. From these seed points (number 0 points in Fig 3), the region can be grew by finding all the neighbouring points (all the points around number 0 point in Fig 3) that have similar properties. In practice, the homogenous points (number 2, 4, and 8 points in the Fig 3) have similar colours or intensities, etc., with the seed points, as illustrated in Fig 3.

**Fig 4:**
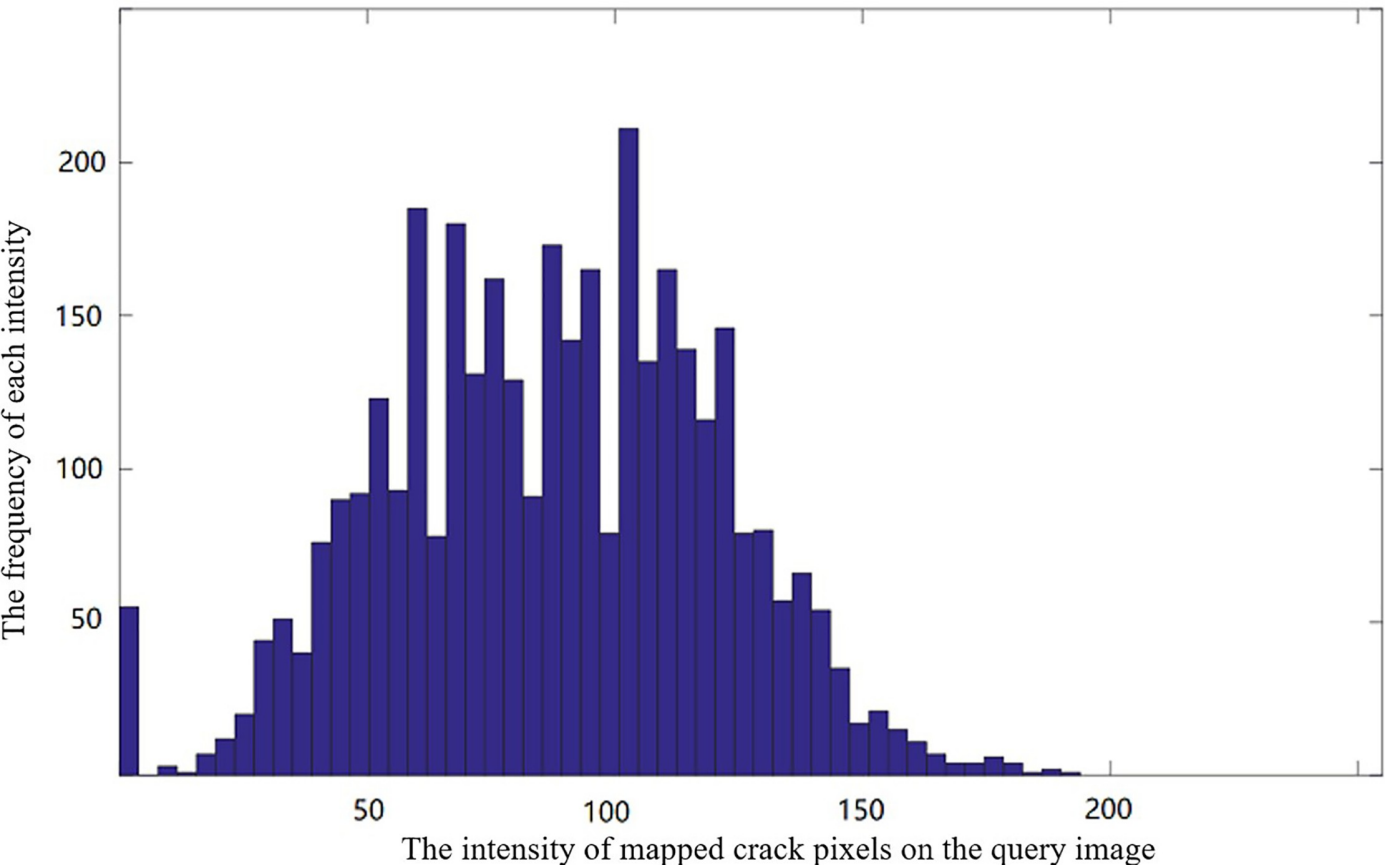
Histogram of pixel intensities from the mapped crack data on the query image.

As the crack image pixels have similar intensity values in the same image, the authors analyse the pixel intensity histogram from the mapped crack pixels. From the mapped label, the pixel values can be computed from the mapped correspondences on the query image. All these pixel values *I*(*μ*',*ν*') associated with the mapped crack label follow certain pattern, as shown in Fig 4. Fig 4 illustrates a typical image intensity distribution of the mapped crack pixel, which demonstrates a Gaussian-like model. It can be used as a constraint for crack growing. Therefore, this paper develops a Gaussian model to fulfil this task. This paper thus utilizes the Gaussian model to represent such distribution. The corresponding mean and standard deviation can be computed as follow:

$$\omega =\sum_{i}\sum_{j}I(i,j)/N$$ (19)

$$\sigma =\sqrt{\sum_{i}\sum_{j}\left(I(i,j\right)-\omega {)}^{2}/N}$$ (20)

where [*i*,*j*]^*T*^∈*Q* and *N* is the number of mapped crack label pixels.

Note that, the proposed method allows that each query crack image has unique pixel Gaussian model. Compared to the hard threshold methods in literature, the proposed method is thus more robust and adaptive for region growing. From the computed Gaussian model, the authors can quickly determine if a neighbour image *I*(*p*_*μ*_,*p*_*ν*_) has the similar properties with the seed points. As crack region in the image usually has low image intensities, the authors can thus set a range of image intensities from the Gaussian model parameters, such as the mean and the standard deviation. Hence, a point (*p*_*μ*_,*p*_*ν*_) is classified into crack if its intensity satisfied the following conditions:

$$I(p_{\mu },p_{v})\in [0,\omega +\lambda \sigma ]$$ (21)

where *λ* is determinate ratio that is empirically set in the practical applications. The detail for selection of determinate radio can be seen is Section 3.2.

As a result, the RGM can be applied to Eq (21) to detect all the neighbour homogenous points near the seed points. By utilizing the RGM, the authors can detect all the pixels belonging newly grown cracks in the query crack image. These newly grown crack pixels are important for us to analyse the crack growth and predict the crack severity.

## 3. Experimental results

**Fig 5:**
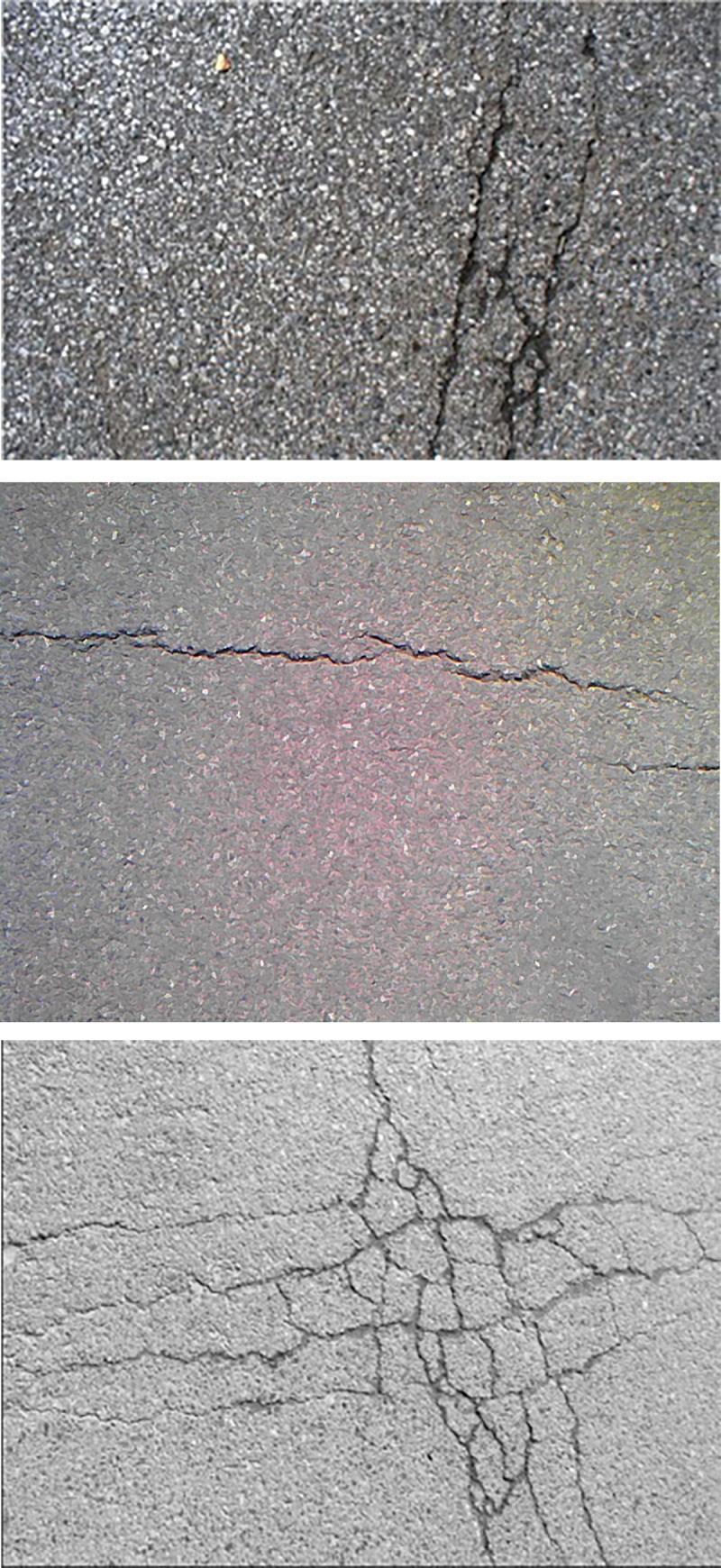
Typical collected pavement crack images.

In order to test the proposed method, two scenarios were selected for experiments. In order to test the proposed method, two scenarios were selected for experiments. The first scenario was Youyi Road near Yujiatou campus of Wuhan university of technology (WUT) (GPS coordinate: 114.363, 30.615). The second one was Linjiang Road, Wuhan city (GPS coordinate: 114.354, 30.621). The total distance for two scenarios is 6.5KM that amount of pavement crack data has been collected. Both scenarios have high traffic volume every day. The types of pavements for two scenarios are asphalt. Various kinds of pavement crack were recorded, such as longitudinal crack, transverse crack and fatigue crack, as illustrated in Fig 5.

**Fig 6:**
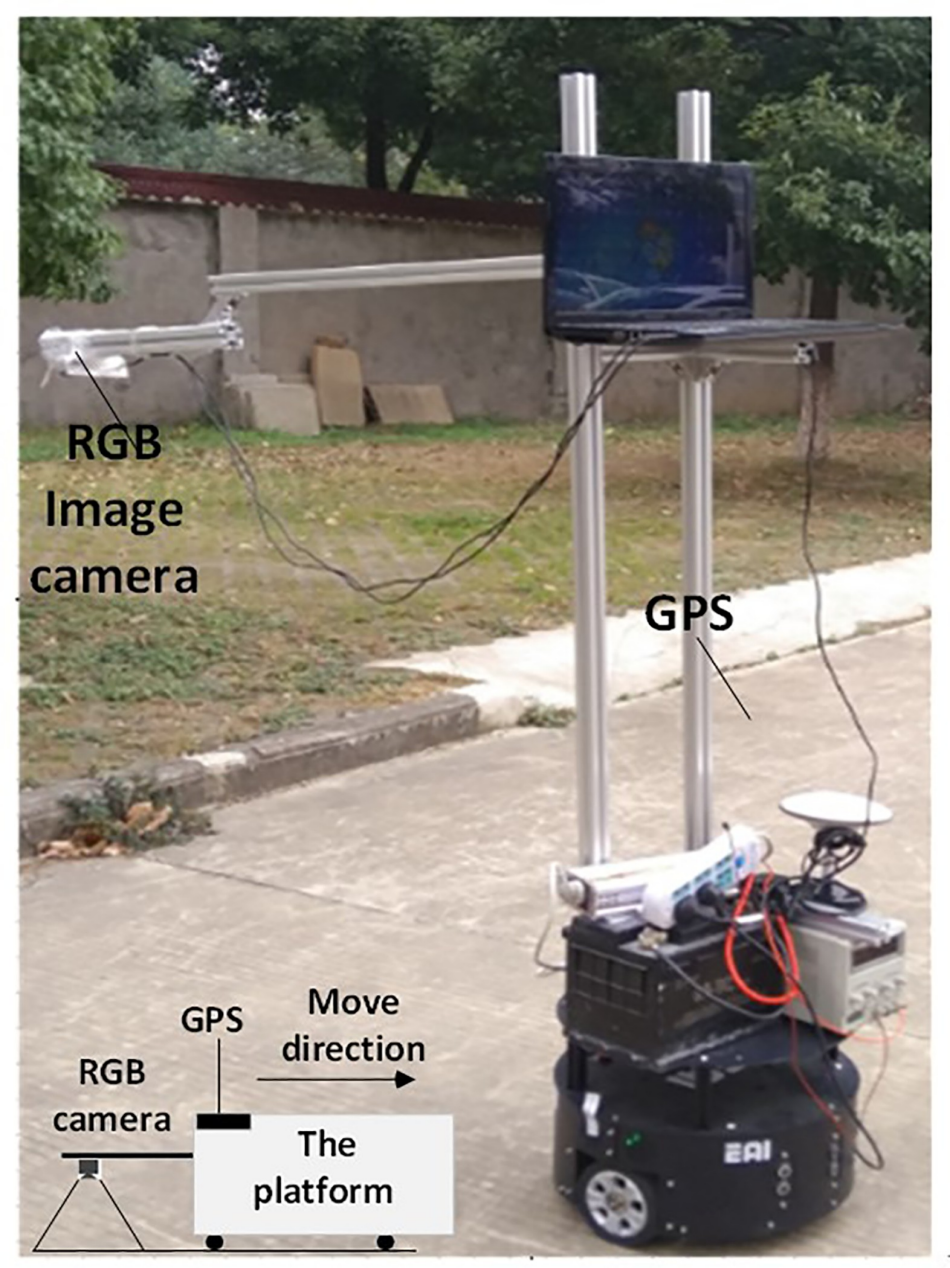
The developed mobile platform for crack data collection.

The experimental data was obtained by a mobile platform (Fig 6), which was equipped with a PointGrey RGB camera and GPS receiver. The image resolution of the RGB camera is 1500×960 (in pixel). The RGB camera faced downward to the ground to capture pavement images. In such setup, the actual physical size of each pixel in the image is 1mm×1mm. Besides RGB camera, a GPS receiver was used to simultaneously collected GPS location with about 2-10m accuracy. The GPS receiver and the RGB camera were synchronized for data collection.

### 3.1. Crack data collection and evaluation

**Fig 7:**
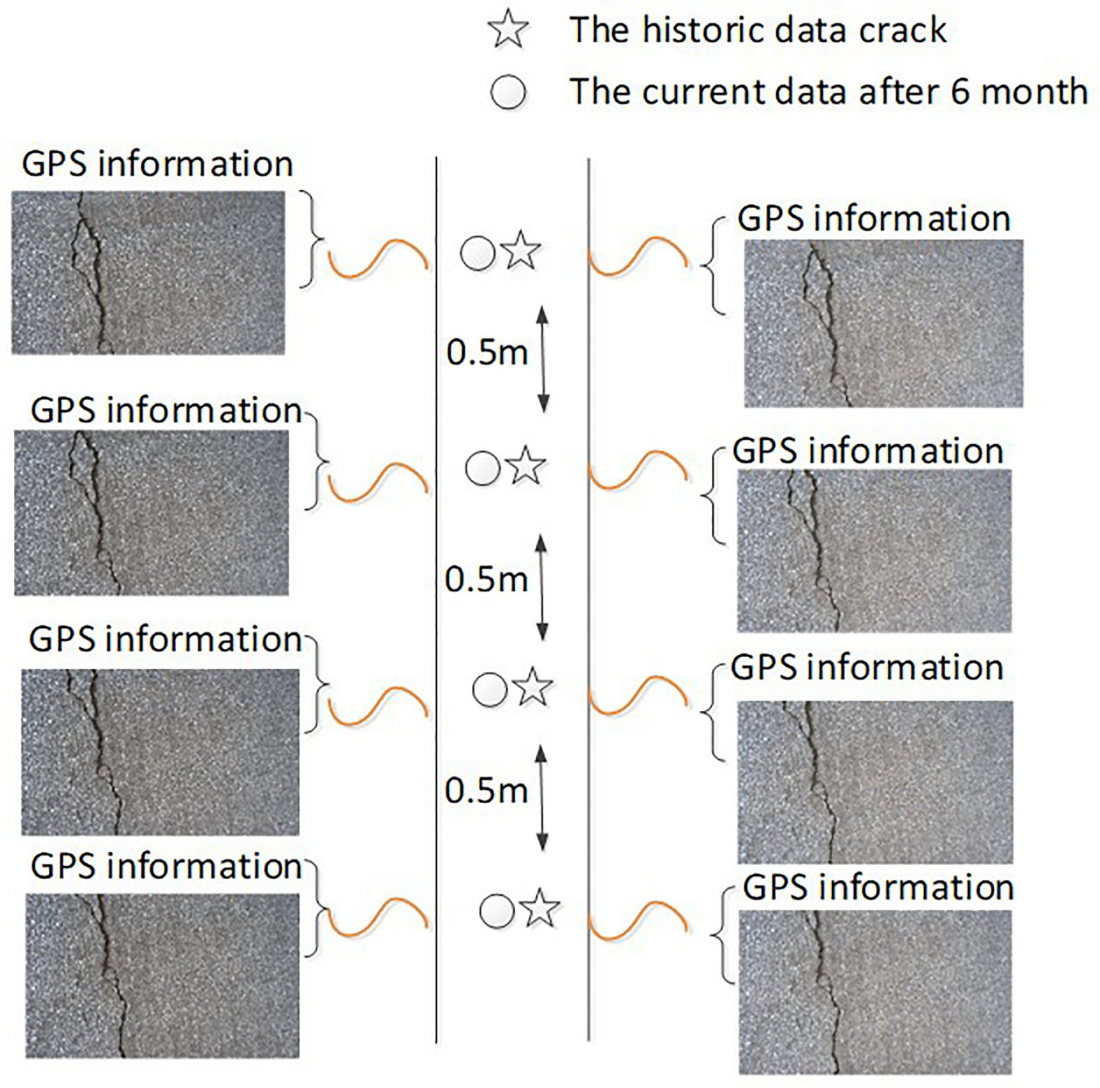
The organization form of query data and historical data.

For the two scenarios, we collected the pavement data in two different times with different lighting and weather conditions. We collected 113 crack images and GPS information as historical data. After 6 months, we collected 113 crack images and GPS information at same place and these data were query data. In these historical pavement images, all the crack pixels were manually labelled. As a result, the pavement images, the labelled crack data, and the associated GPS data, were organized to formulate the reference crack data, as shown in Fig 7. The data collected later were used as the query data. In both historical and query data, the distance between the data collection was 0.5 meter.

In order to evaluate the performance of the proposed reference crack based method, the ground truth data were first obtained in a manual way. Hence, the crack detection results were compared to the ground truth to identify true crack pixels (as TP), false crack pixels (as FP), and true non-crack pixels (as TN), and false non-crack (as FN). Based on the TP, FP, TN, FN, the authors employ the Precision *P*, Recall *R*, and F-Measure as criterion to evaluate the performance of the proposed method as follow:

$$P=\frac{TP}{TP+FP}$$ (22)

$$R=\frac{TP}{TP+FN}$$ (23)

$$F-Measure=2\times \frac{P\times R}{P+R}$$ (24)

### 3.2. Multi-scale localization and determinate radio selection

**Fig 8:**
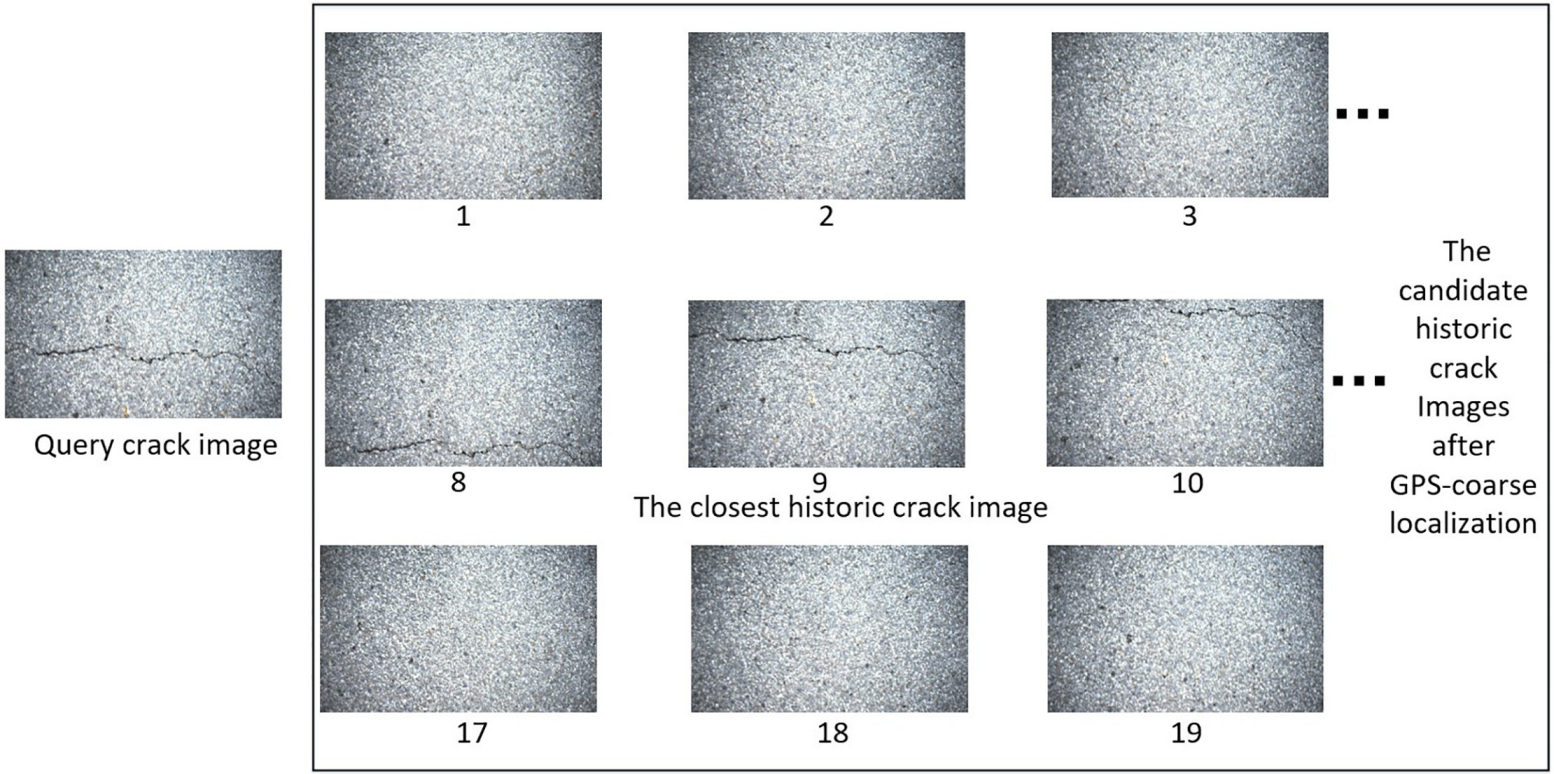
The results for GPS-based coarse localization. The image-level localization by matching local feature points and the number of matched feature point pairs for different historical images are shown.

In this section, the authors demonstrated the experimental results for multi-scale localization and determinate radio selection. Multi-scale localization consists of GPS-based coarse localization, image-level localization, and pixel-level localization. The results for GPS-based coarse localization are illustrated in Fig 8. The accuracy of GPS localization is close to 10 meter. Therefore, the number for candidate historical crack images after GPS-based coarse localization is 19. As can be observed in Fig 8, the “closest” historical crack image in the candidate historical crack images database is No.9. And the authors try to automatically determine the No. 9 image by using the proposed image-level localization method.

**Fig 9:**
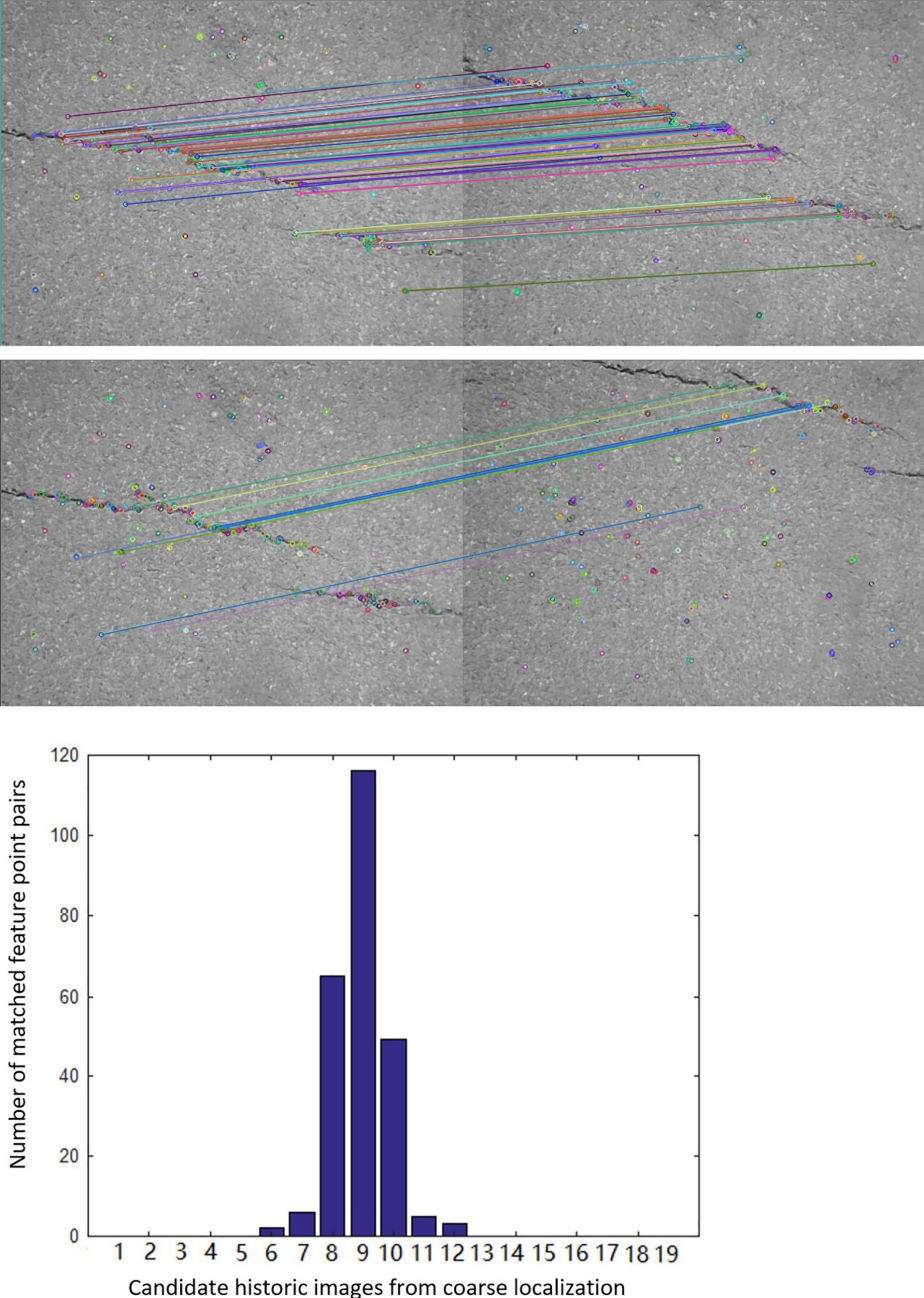
The local feature point pairs for query crack image and candidate historical crack images.

In order to illustrate the multi-scale localization, the authors demonstrated the number of local feature point pairs between query crack image and each candidate historical crack image. The local feature point pairs are illustrated in Fig 9A. The local feature point pairs increase with the distance that between query crack image and historical crack image decrease. It can be seen in Fig 9A. The number of local feature point pairs for query crack image and No.9 historical crack image is 117. The number of local feature point pairs for query crack image and No.10 historical crack image is 54. The former has more local feature point pairs comparing with the latter. By this way, we compared the number of local feature point pairs between query image and each historical image. As illustrated in Fig 9B, No.9 candidate historical crack image approximates most to query crack image, which confirm to the ground truth.

**Fig 10:**
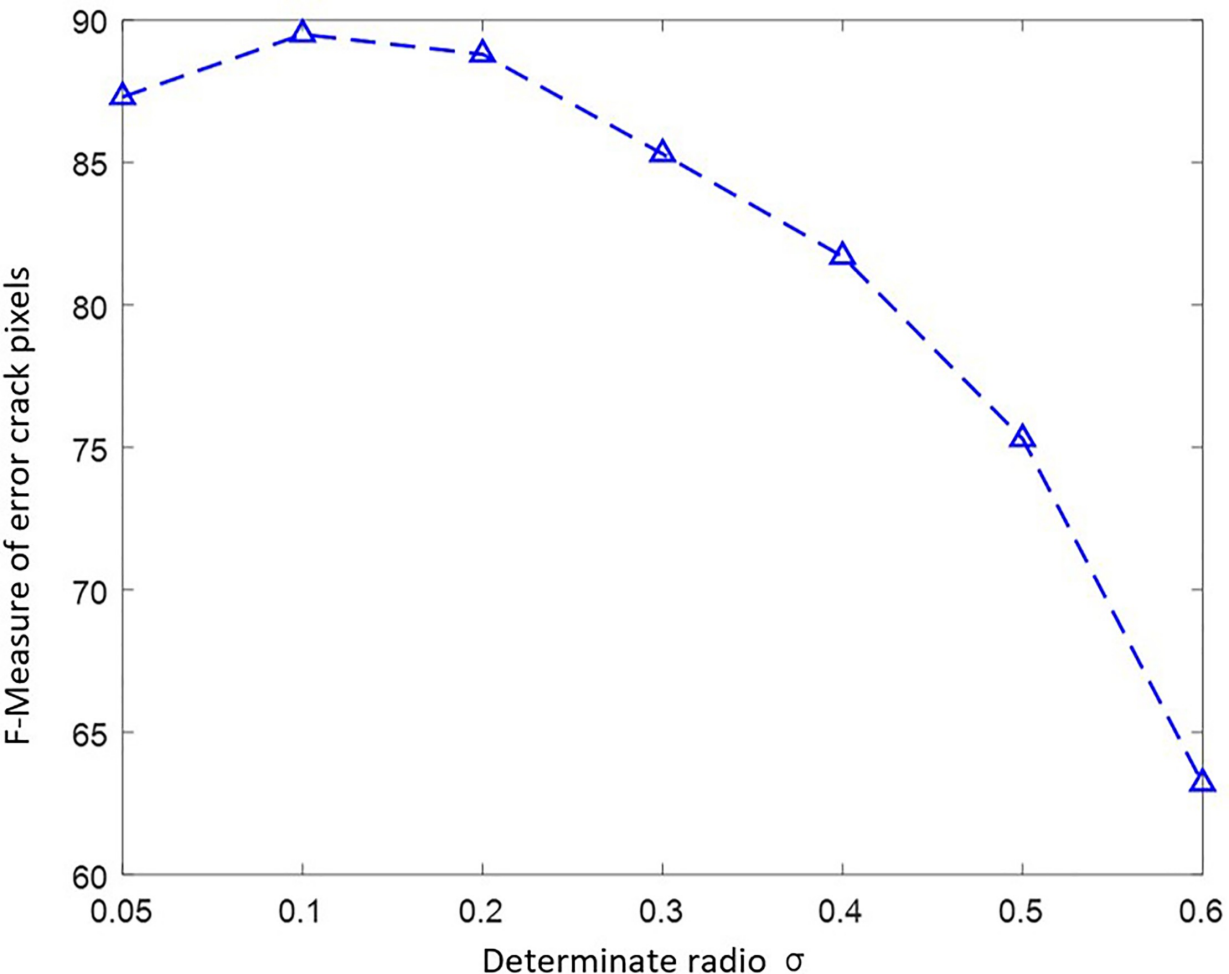
F-Measure of error crack pixels for different determinate radio.

After mapping historical labelled crack images onto the current crack image by image correspondence transform, the RGM can be utilized to detect the crack growing. This paper selected different determinate radio *λ* to control crack growing. The determinate radio are 0.05, 0.1, 0.2, 0.3, 0.4, 0.5, and 0.6, respectively. The F-Measure of error crack pixels for different determinate radio are illustrated in Fig 10.

As illustrated in Fig 10, F-Measure of error crack pixels for different determinate radio is a convex curve. When determinate radio 0<*λ*<0.1, the F-Measure increase with determinate radio increase. When determinate radio 1>*λ*>0.1, the F-Measure become decrease with determinate radio increase. The peak value of determinate radio for F-Measure is *λ* = 0.1. It indicates that determinate radio *λ* = 0.1 has best performance in crack growing detection. Therefore, the determinate radio *λ* = 0.1 is selected to test the crack growth.

### 3.3. Crack detection and analysis on WUT campus

Firstly, the authors tested the proposed method on WUT campus dataset. Various kinds of pavement cracks, such as longitudinal crack, transverse crack and fatigue crack, were tested in the experiment. Due to time interval between historical crack and query crack is 6 month and huge vehicle flowrate in the test road, it is enough for crack growth. The ground truth of crack grown pixels was obtained by human annotated. In this experiment, the authors utilized the precision, recall, and F-Measure as criterion to evaluate the proposed method. The contrast experimental results for three methods are illustrated in Table 1.

**Table 1:** Crack growth experiment results for three methods.

Method	Precision	Recall	F-Measure
The proposed method	95.4%	84.3%	89.5%
CrackTree Method [23]	80.1%	91.3%	85.3%
Seg-Ext Method [24]	58.3%	64.2%	61.1%
FCN Method [25]	82.7%	78.2%	80.4%

As illustrated in Table 1, the precision, recall, and F-Measure for the proposed method were 95.4%, 84.3%, and 89.5%. In addition, the authors compared the proposed method with other crack detection methods. The contrast methods detect the newly crack pixels by comparing the detection result of historical crack image and query crack image for twice. The contrast experimental results also can be seen in Table 1. As illustrated in Table 1, The precision, recall, and F-Measure were 80.1%, 91.3%, and 85.3% for the CrackTree [23], respectively. The precision, recall, and F-Measure was 58.3%, 64.2%, and 61.1% for Seg-Ext [24]. The precision, recall, and F-Measure were 82.7%, 78.2%, and 80.4% for FCN [25]. The seed points, which are mapped from historical crack pixels, have high confidence value for crack pixels. Therefore, the result of growth crack pixels detection, which through analysed from seed points, is more accuracy than detecting grown crack in the image directly. The result also shows the proposed method has good performance for crack growth detection.

**Fig 11:**
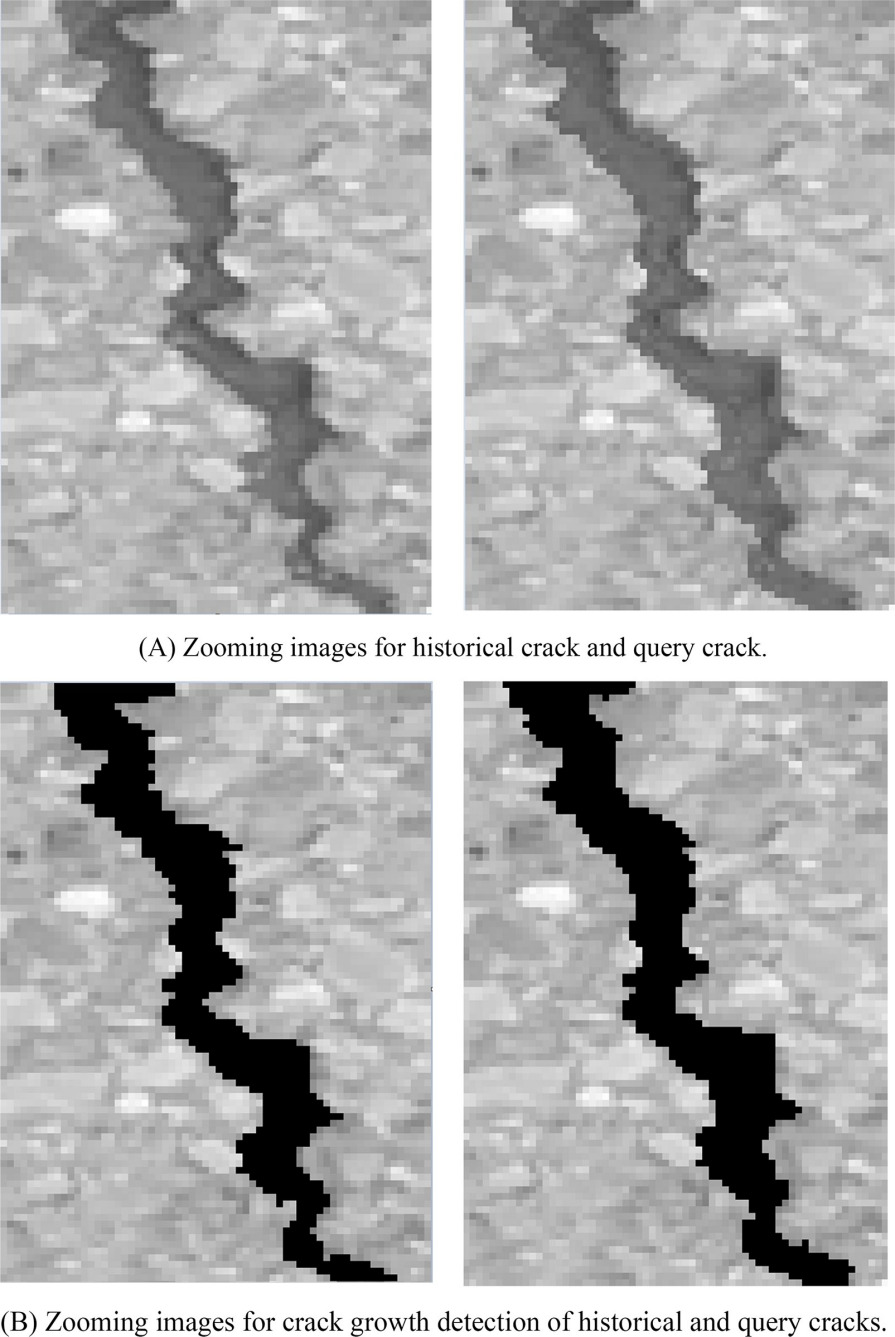
Crack growth detection on WUT dataset.

**Fig 12:**
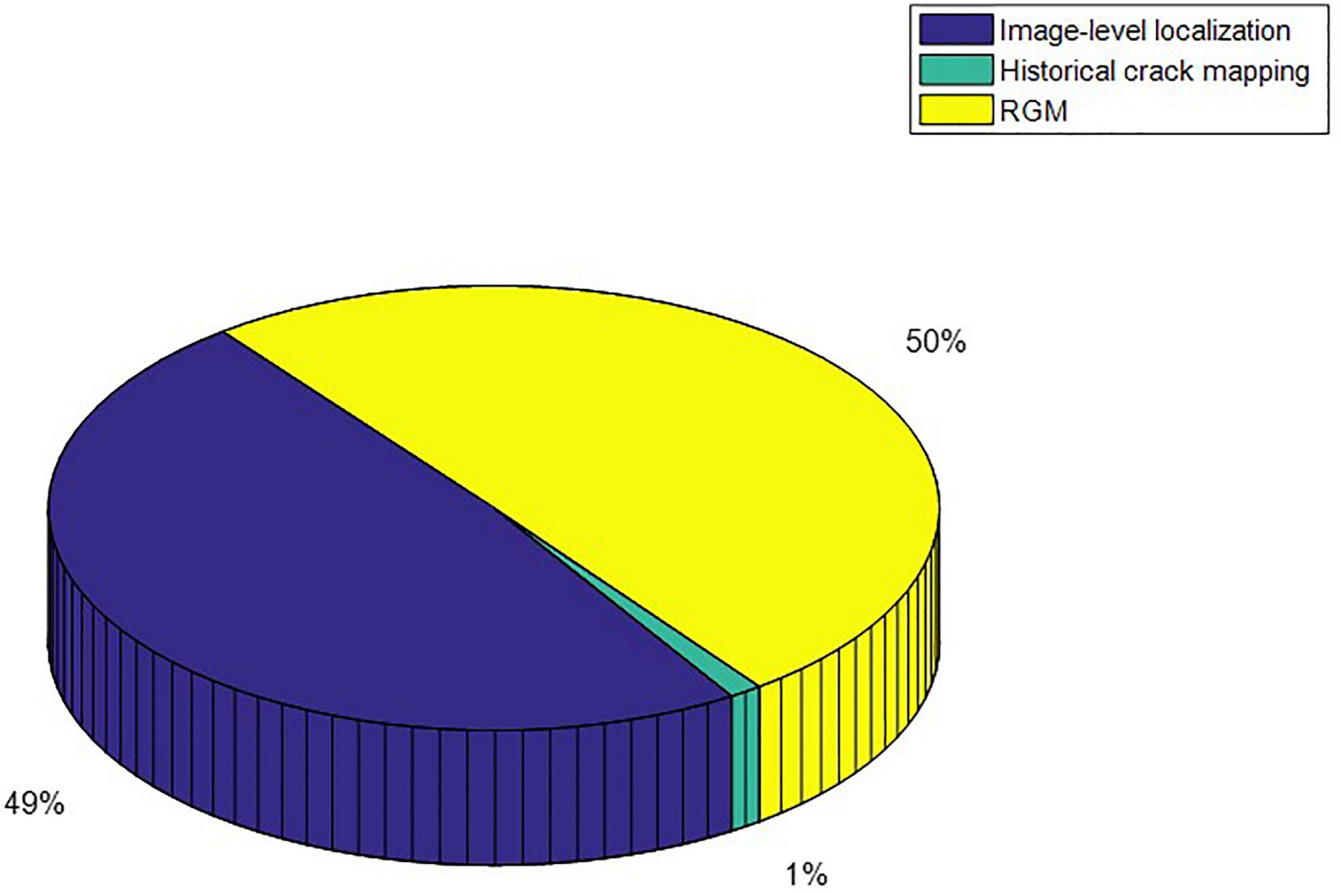
Time consumption of the proposed method.

The results of crack growth on WUT dataset are illustrated in Fig 11. The zooming images for historical crack and query crack are illustrated in Fig 11A and corresponding crack growth detection images are illustrated in Fig 11B. Due to the randomness of the crack growth, the growth crack pixels are surround to the previous pavement crack pixels. According to Fig 11, the ground truth for the crack growth were 135 pixels and there were 141 pixels for crack growth detection. The slight crack growth still can be detected by utilizing proposed method. Time consumption of each procedure of proposed method is illustrated in Fig 12. Since the step of image-level localization is time-consumption, the average time consumption of procedure of image-level localization is 273ms. The procedure of historical crack mapping is the procedure of matrix transformation so that the time consumption is 7ms. The procedure of RGM need to search the satisfied pixels that near crack pixels. It is time-consuming step and the time consumption is 293ms. The average time consumption of whole procedure is 583ms. It illustrates that the proposed method is effective for crack growth detection.

### 3.4. Crack detection and analysis on Linjiang Road

The authors also tested the proposed crack growth analysis on Linjiang Road dataset. Same with WUT dataset, various crack such as longitudinal crack, transverse crack and fatigue crack has been tested in the experiment. The authors also selected the tested road with huge vehicle flowrate. Moreover, time interval between historical crack and query crack is also 6 month. Therefore, it is enough for crack growth. The ground truth of crack grown pixels also can be obtained by human annotated. In the experiment, the authors utilized the precision, recall, and F-Measure as criterion to evaluate the proposed method. The contrast experiment results for three methods are illustrated in Table 2.

**Table 2:** Crack growth experiment results for three methods.

Method	Precision	Recall	F-Measure
The proposed method	94.3%	82.9%	88.2%
CrackTree Method[23]	78.3%	89.9%	83.7%
Seg-Ext Method ([24]	55.9%	63.4%	59.7%
FCN Method [25]	81.9%	77.5%	79.6%

As illustrated in Table 2, the precision, recall, and F-Measure for the proposed method were 94.3%, 82.9%, and 88.2%. In addition, the authors compared the proposed method with other crack detection methods. The contrast experimental results also can be seen in Table 2. As illustrated in Table 2, The precision, recall, and F-Measure were 78.3%, 89.9%, and 83.7% for the CrackTree [23], respectively. The precision, recall, and F-Measure were 55.9%, 63.4%, and 59.4% for Seg-Ext [24]. The precision, recall, and F-Measure were 81.9%, 77.5%, and 79.6% for FCN [25]. The seed points, which are mapped from historical crack pixels, have high confidence value for crack pixels. Therefore, the result of growth crack pixels detection, which through analysed from seed points, is more accuracy than detecting grown crack in the image directly. The experimental results of Youyi Road dataset are higher than the results of Linjiang Road dataset. The reason is that pavement of Linjiang Road is worse than Youyi Road. Even so, it still has a good performance without shape decline. It shows the robustness of proposed method. The contrast experimental results also showed the proposed method has good performance for crack growth detection.

**Fig 13:**
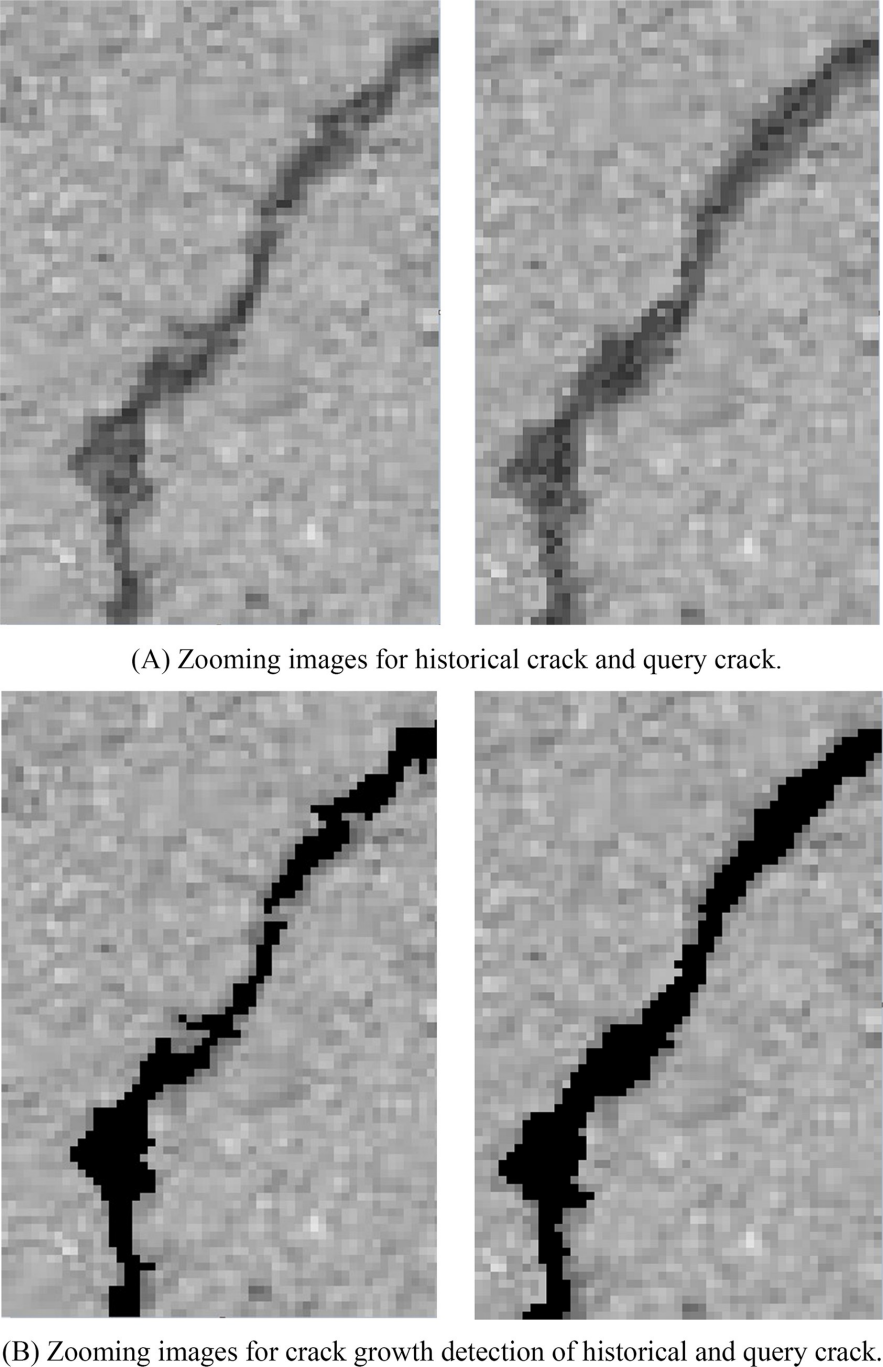
Crack growth on Linjiang Road dataset.

**Fig 14:**
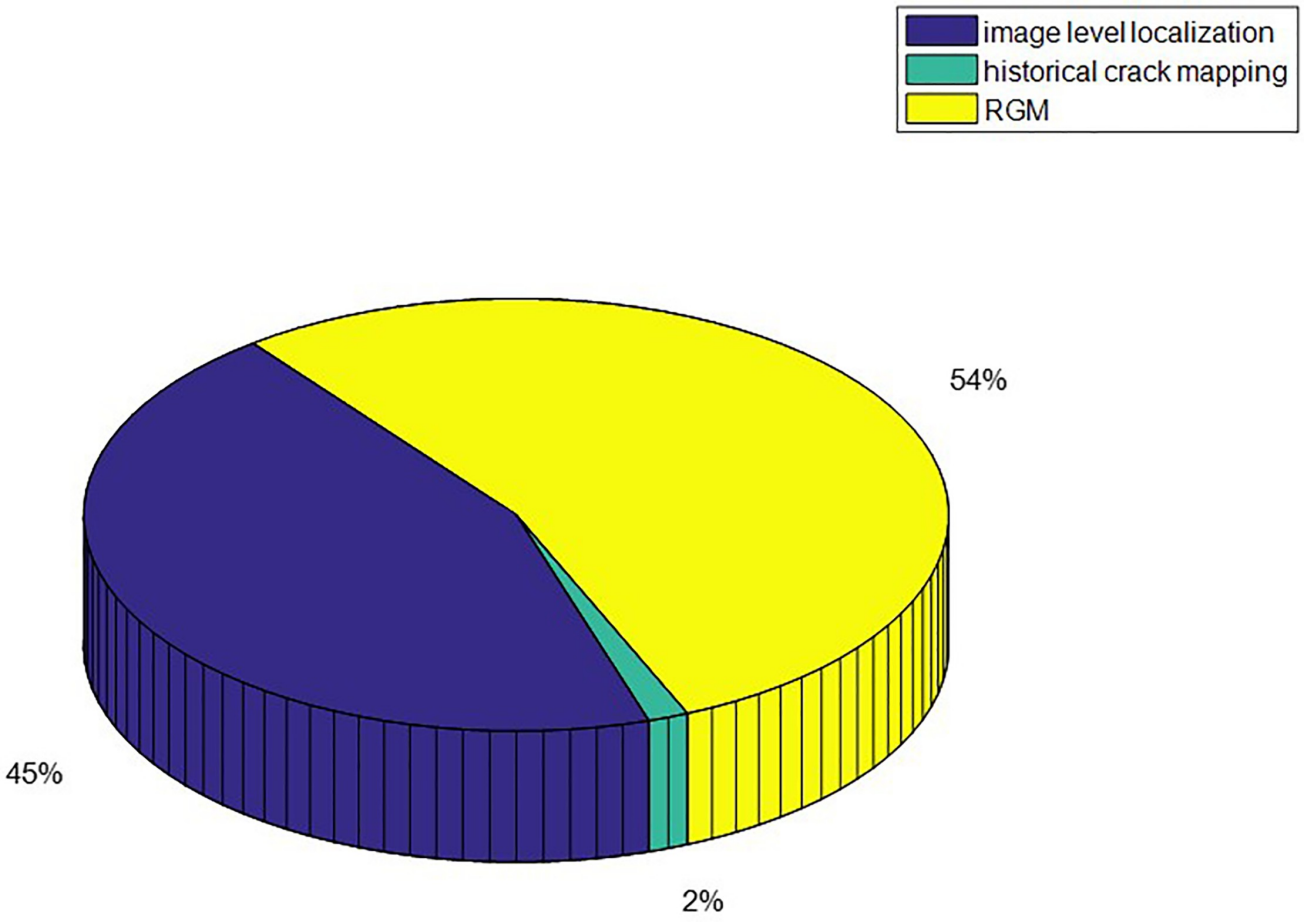
Time consumption of the proposed method.

The results of crack growth on Linjiang Road dataset are illustrated in Fig 13. The zooming images for historical crack and query crack are illustrated in Fig 13A and corresponding crack growth detection images are illustrated in Fig 13B. Due to the randomness of the crack growth, the growth crack pixels are surround to the previous pavement crack pixels. According to Fig 13, the ground truth for the crack growth were 189 pixels and there were 193 pixels for crack growth detection. The slight crack growth still can be detected by utilizing proposed method. The time consumption of each procedure of proposed method is illustrated in Fig 14. Since the step of image-level localization is time-consumption, the average time consumption of procedure of image-level localization is 294ms. The procedure of historical crack mapping is the procedure of matrix transformation so that the time consumption is 10ms. The procedure of RGM need to search the satisfied pixels that are closed to crack pixels. It is time-consuming step and the time consumption is 356ms. The average time consumption of whole procedure is 660ms. It illustrates that the proposed method is effective for crack growth detection.

## 4. Conclusions

This paper evidently demonstrates that historical crack data could greatly enhance pavement crack analysis. In order to refer to historical crack data, the core is to establish the image correspondences between the current and historical crack images, a step called localization in this paper. The authors proposed a multi-scale localization strategy for image correspondences. It consists of a coarse localization from GPS matching, image-level localization from visual feature matching, and finally pixel-level localization from accurate in-vehicle camera calibration. The multi-scale localization allows us to predict query crack by using historical crack data, therefore can greatly improve the performance of crack detection and recognition. The proposed method has been validated by using the actual pavement data collected before and after 6 months with the earlier crack data as the reference. The results for crack analysis are promising in term of reliability and accuracy. The paper suggests a novel strategy to pavement crack analysis in support of modern pavement management systems.
